# Modulation of the gut microbiota by nutrients with prebiotic properties: consequences for host health in the context of obesity and metabolic syndrome

**DOI:** 10.1186/1475-2859-10-S1-S10

**Published:** 2011-08-30

**Authors:** Nathalie M Delzenne, Audrey M Neyrinck, Patrice D Cani

**Affiliations:** 1 Université catholique de Louvain, Louvain Drug Research Institute, Metabolism and Nutrition Research Group, Brussels, Belgium

## Abstract

The gut microbiota is increasingly considered as a symbiotic partner for the maintenance of health. The homeostasis of the gut microbiota is dependent on host characteristics (age, gender, genetic background…), environmental conditions (stress, drugs, gastrointestinal surgery, infectious and toxic agents…). Moreover, it is dependent on the day-to-day dietary changes. Experimental data in animals, but also observational studies in obese patients, suggest that the composition of the gut microbiota is a factor characterizing obese versus lean individuals, diabetic versus non diabetic patients, or patients presenting hepatic diseases such as non alcoholic steatohepatitis. Interestingly, the changes in the gut microbes can be reversed by dieting and related weight loss. The qualitative and quantitative changes in the intake of specific food components (fatty acids, carbohydrates, micronutrients, prebiotics, probiotics), have not only consequences on the gut microbiota composition, but may modulate the expression of genes in host tissues such as the liver, adipose tissue, intestine, muscle. This in turn may drive or lessen the development of fat mass and metabolic disturbances associated with the gut barrier function and the systemic immunity. The relevance of the prebiotic or probiotic approaches in the management of obesity in humans is supported by few intervention studies in humans up to now, but the experimental data obtained with those compounds help to elucidate novel potential molecular targets relating diet with gut microbes. The metagenomic and integrative metabolomic approaches could help elucidate which bacteria, among the trillions in human gut, or more specifically which activities/genes, could participate to the control of host energy metabolism, and could be relevant for future therapeutic developments.

## Introduction

Overweight and obesity have reached epidemic levels. In 2008, 1,5 billion adults were overweight and over 200 million men and nearly 300 million women were obese. Worldwide obesity has more than doubled since 1980. Overweight and obesity are defined as an excess of fat mass but is often assessed by the calculation of the body mass index (BMI), calculated by dividing the weight (in kg) by the size^2^ (in meter^2^). Subjects with a BMI greater than or equal to 25 or 30 kg/m^2^, are considered as overweight and obese, respectively. [1].

Obesity is associated with a cluster of metabolic disorders such as insulin resistance, type 2 diabetes, fatty liver disease, atherosclerosis, hypertension and stroke but also with cancer, asthma, sleep apnoea, osteoarthritis, neuro-degeneration, and gall-bladder disease [2]. The factors included in the definition of the metabolic syndrome are dysglycemia, raised blood pressure, elevated triglyceride levels, low high-density lipoprotein cholesterol levels and obesity (particularly central adiposity). The presence of any three of these five risk factors constitutes a diagnosis of metabolic syndrome [3].

**Figure 1:**
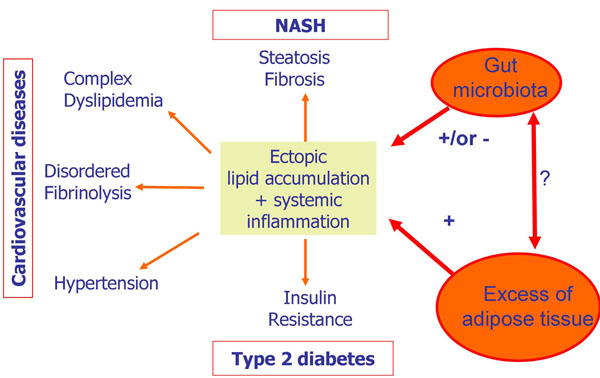
**Obesity and related metabolic diseases: which role for the gut microbiota?** In several organs and in the serum, lipid accumulation and pro-inflammatory processes contribute to metabolic alterations leading to an increased risk of cardiovascular disease, diabetes and non alcoholic steato-hepatitis. The production of metabolites and cytokines by the adipose tissue is playing a crucial role. A novel partner to consider would be the gut microbiota which could, depending on its composition and activity, either positively or negatively contribute to metabolic syndrome. NASH, Non-Alcoholic SteatoHepatitis

The major cause of obesity is a positive energetic balance resulting from an increased energy intake from the diet and a decreased energy output associated i.e., with low physical activity. In addition, the genetic background participates to the inter-individual difference in term of energy expenditure and storage capacity. However, growing evidence suggests that among the “external” factors contributing to the host response towards nutrients, the gut microbiota represents an important one. We are harbouring 10^14^ bacteria in our gut; the gut microbiome (microbial genome) represents therefore 150 fold more gene than the human genome, and the metabolic potential of the gut microbiota, analysed for example in the European MetaHit project, is tremendous [4,5]. Even if most of the functions of the microbial genes are still unknown, and if we are conditioned at birth with a “personal” profile of gut microbes, experiments performed in a model of mice colonized with the human gut microbiota revealed that changes in the diet composition (from high carbohydrates to western diet which caused the mice to become obese) allowed a rapid switch of the microbial community [6]. Interestingly, this modified gut microbiota could be transferred to germ free mice concomitantly with the obese phenotype [6]. Those data suggest, as illustrated in figure 1, that the gut microbiota composition/activity is a factor to take into account when assessing the risk factors related to obesity, but also to associated diseases, such dyslipidemia and atherosclerosis, insulin resistance and diabetes, hepatic steatosis and steatohepatitis [5,7-10].

The purpose of this review is to present the underlying mechanisms by which nutrients and food components which target the gut microbiota may play a role in the management of obesity and metabolic syndrome. This paper will mostly focus on the data relating the effect of non digestible carbohydrates exhibiting prebiotic properties, such as dietary fructans.

## Gut microbiota composition and metabolism associated with the metabolic disorders in obese individuals

The gut microbiota present particular genetic and metabolic attributes, thereby giving the host the potential to live in symbiosis with those “external” cells which are ten fold more numerous than the number of cells in the human body [8,11-13].

Novel culture-independent technologies based on the analysis of the bacterial 16S rRNA gene (e.g., pyrosequensing) allowed significant progress in the knowledge of our microbial partners with which we live in symbiosis [14,15]. Most of the studies concerning the relation between host phenotype involving obesity and/or diabetes and gut microbiota, report changes of the relative abundance of phyla, gender, or species of bacteria, which correlate – positively or negatively- with biomarkers of the disease measured in the host (body mass index, glycemia, pro-inflammatory cytokines….) [10,16]. The first study describing qualitative changes of the gut microbiota in human obese individuals was published a few years ago [17]. In this study, the analysis of fecal samples of obese versus matched lean individuals showed a shift in bacterial phyla (lower Bacteroidetes and more Firmicutes) [17]. Interestingly, the authors observed that after weight loss (following a fat restricted or a carbohydrate restricted low calorie diet), the ratio of Bacteroidetes to Firmicutes approached a lean type profile after 52 weeks [17]. The focus on Bacteroidetes seems to be controversial. A reduction of Bacteroidetes community in obese patients was confirmed later [18]. In another study, no differences were detected between obese and non-obese individuals in term of the proportion of Bacteroidetes measured in the fecal samples, and no significant changes of the percentage of Bacteroidetes occurred in feces from obese subjects upon weight loss [19]. In accordance with these data, even more Bacteroidetes were detected in a group of obese subjects than in normal-weight individuals [20]. A subgroup of Bacteroidetes (*Prevotellaceae*) was significantly enriched in the obese individuals. Moreover, the same authors showed that surgical treatment for morbid obesity (gastric bypass) strongly increased *Gammaproteobacteria* (members of the family *Enterobacteriaceae*) and proportionally decreased Firmicutes [20]. The methodology used for bacterial analysis could explain certain discrepancies between results published by different groups [21].

Recently, three robust clusters referred to as “enterotypes” have been identified in individuals from different countries and a continent [22]. They found that these enterotypes were identified by the variation at the level of one of the three following genera : *Bacteroides*, *Prevotella* and *Ruminococcus.* It is important to note that these enterotypes were not correlated with the host characteristics such as body mass index (BMI), age, gender or nationality. Importantly, this large study did not revealed any correlation between BMI and Firmicutes/Bacteroidetes ratio [22]. However, although no difference in the microbiota composition has been described, a set of 3 genes modules have been strongly correlated with the host’s BMI, two of which are ATPase complexes, supporting the link found between the gut microbiota’s capacity for energy harvest and obesity in the host [22].

The hypothesis of more specific modulation of the gut microbiota community in obesity (instead of those obtained at the wide *phylum* levels) is supported by several studies. *Bifidobacterium* spp. number was higher in children who exhibited a normal weight at 7 years than in children developing overweight [23]. More importantly they observed that the *Staphylococcus aureus* levels were lower in children who maintain a normal weight than in children becoming overweight several years later. The authors proposed that *S. aureus* may act as a trigger of low-grade inflammation [24], contributing to the development of obesity [25]. In agreement with these last findings, significant differences have been observed in gut microbiota composition according to the body weight gain during pregnancy [26]. Interestingly, the authors found significantly higher numbers of *Bacteroides* group in women with excessive weight gain upon pregnancy. They also established a positive correlation between the number of *Bacteroides*, *Clostridium and Staphylococcus* on the one hand, and the weight and BMI before pregnancy, on the other hand. The *Bifidobacterium* genus was present in higher numbers in normal-weight than in overweight women and also in women with lower weight gain during pregnancy [26]. The *Bifidobacterium* genus was also poorly represented in the fecal samples of diabetic patients compared with healthy individuals [27]. Nevertheless, a recent report has shown that weight loss could also be associated with reduced level of *Bifidobacterium bifidum* and *Bifidobacterium breve* counts and increased *Bifidobacterium catenulatum*[28]. The level of *Bifidobacterium* genus is also decreased upon weight loss after bariatric surgery performed in obese individuals [29]. Indeed, *Bifidobacterium spp.* represent an important and complex group of bacteria whose presence is often associated with beneficial health effects [30-32]. Another interesting bacterial species is *Faecalibacterium prausnitzii*, whose level is decreased in subjects with diabetes versus non diabetic obese, and which is associated negatively with inflammatory markers measured in the serum of obese individuals before and after roux- and Y gastric bypass surgery [29].

Other selective changes of bacterial composition have been described in obese human individuals, for which the relation with fat mass are sometimes controversial. Lactobacilli counts were higher in 8 out of 20 obese patients compared to the number measured in lean individuals (detected in only 1 out of 20) [18]. Paradoxically, the weight loss due to calorie restriction and physical activity in overweight adolescents also increases lactobacilli [28]. The focus on lactobacilli level may come from the fact that they are part of the Firmicutes phyla, which is increased upon obesity in most of the published studies. It seems, however, that the targeted probiotic approach with specific strains of Lactobacillus does not increase, but even decreases the metabolic alterations occurring in obesity, even if the number of interventional studies in humans, reported until now, remains low. The administration of *Lactobacillus gasseri* has been shown to decrease fat mass (visceral and subcutaneous) and body mass index in obese and type 2 diabetic patients [33]. The administration of *Lactobacillus* spp. has been shown to positively impact on insulin sensitivity [34]. They showed, in a double blind randomised study performed in 45 male subjects with type 2 diabetes, impaired, or normal glucose tolerance, that the administration of *L. acidophilus* NCFM for four weeks, preserved the insulin sensitivity -assessed by an euglycemic hyperinsulinemic clamp-, whereas it decreased in the placebo group [34]. Finally, the early gut microbiota modulation with probiotics (*Lactobacillus rhamnosus* GG and *Bifidobacterium lactis* Bb12) reduced the body mass index in young children by restraining excessive weight gain during the first years of life (from 0 to 10 years of follow-up) [35]. The probiotic approach in experimental studies in animals also suggests a decrease in adiposity in different animal models of obesity. For example, the treatment of high fat fed mice with specific strains of *Lactobacillus plantarum* or *Lactobacillus paracasei spp paracasei* reduced adipocyte cell size and body fat [36,37]. More recently, it was shown that rats fed with high-energy-dense diet at pregnancy, throughout lactation and until 6 months of age, exhibited a lower weight gain, and a lower retroperitoneal adipose tissue when supplemented with *Lactobacillus plantarum*, an effect not observed when treated with *Escherichia coli*[38].

Those data suggest that specific changes in the gut microbiota characterize the obese state and associated metabolic diseases, including diabetes. However, we may not conclude, from the papers published until now, that targeting one specific bacterial target is sufficient to get an improvement of a complex disease such as obesity. Only few intervention studies have been performed with selected probiotics, and this had never been performed in order to counteract a drop in a specific “indigenous” bacteria considered as beneficial.

## Gut microbes/nutrients interactions: a way to produce bioactive metabolites prone to interact with host tissues

It is becoming feasible and relevant to focus on the metabolic and functional properties of the gut microbiota. This approach reveals, in certain studies, that the changes in the gut microbiota composition occurring in obese mice (*ob/ob* mice or high fat diet fed mice) could be in favour of bacteria exhibiting saccharolytic activities [39-41]. The studies performed in germ free mice colonized with the caecal content obtained from obese and insulin-resistant mice have shown that the phenotype (obesity, insulin resistance and/or inflammation) can be transferred to the germ-free recipient through the microbial colonization, thereby suggesting that some elements of the gut microbes drive metabolic alterations in host tissues [40,42]. The experiments performed on germ free mice colonized with gut microbes, and then exposed to different types of diets, are illustrating the fact that the host response in term of fat mass development depends on the interactions between gut microbes and nutrients (for review, see [43]). Recently, it has been shown in obese and lean human individuals that the increase in calorie intake (from 2400 to 3400 kcal/d) promoted rapid changes in the gut microbiota (20% increase in Firmicutes and a corresponding decrease in Bacteroidetes). This was associated with an increased energy harvest of ≈150 kcal, the overfeeding in lean individuals being accompanied by a greater fractional decrease in stool energy loss [44].

The promoting effect of the gut microbiota on intestinal glucose absorption has been suggested in germ free mice colonized with the saccharolytic *Bacteroides thetaiotaomicron* : this effect supports the fact that events occurring upon bacterial fermentation may have an effect in the upper part of the gut, and thereby affecting digestible carbohydrates availability [45]. Conventionalization of germ free mice has been demonstrated to promote body weight gain and fat mass development [46]. Interestingly, the conventionalization also brought about a general increase in the activity of the enzyme lipoprotein lipase (LPL), catalyzing the release of fatty acids and triacylglycerol from circulating lipoproteins in muscle, and adipose tissue. The authors proposed that such an increase was the consequence of suppression of the Fasting-Induced Adipose Factor (FIAF) in the gut. Given that FIAF inhibits the LPL activity, the blunted FIAF expression in conventionalized germ free mice could thus participate to the accumulation of lipids in the adipose tissue [46]. In another set of experiment the authors maintained germ free mice or conventionalized mice on a high fat/high carbohydrates diet (western diet). They found that germ free mice were resistant to the high-fat diet-induced body weight and fat mass gain than the conventionalized mice [47]. This increase in fat mass upon high fat diet feeding in conventionalized versus germ free animals, supports the fact that the fat storage is favoured by the presence of the gut microbiota [46,47]. However, the observation of the metabolic response to high fat diet of germ free versus conventionalized mice, suggest that the presence of carbohydrates in the diet may modulate the development of obesity upon colonization of the gut [48].

Just as the gut microbes influence the metabolic feature of nutrients, it is also clear that the ingestion of specific nutrients play a role in the microbial changes. We have previously demonstrated that diet-induced obesity (high fat-low carbohydrates diet) in mice markedly affects the gut microbial community whereby the levels of *Bifidibacterium* spp. and *E. rectale/Cl. coccoides* group were significantly reduced [25,49]. The effect of feeding a high fat diet to conventional mice and RELMb knock-out mice, the latter of which are resistant to fat-induced obesity, resulted in a decrease in Bacteroidetes and an increase in Firmicutes and Proteobacteria for both genotypes, indicating that high fat diet itself rather than the obese state may account for the changes in microbiota composition [50].

**Figure 2:**
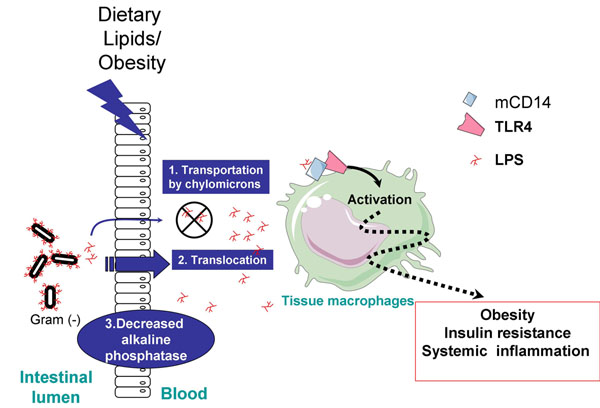
**Occurrence of entotoxemia upon high fat diet feeding and obesity.** An increase level of lipopolysaccharides (LPS) characterizes obese and diabetic individuals. This effect is mostly due to processes involving the transport of LPS from the gut to the blood, those processes being an increase in chylomicron-driven transport of LPS, a rupture of the gut barrier integrity leading to abnormal gut permeability, and a decrease in processes involved in intestinal LPS degradation (alkaline phosphatase activity). The increase in LPS (endotoxemia) thereby activates the macrophages in the different tissues leading to a low tone inflammation involved in the metabolic alterations occurring upon obesity.

Another bacterial element to take into account when assessing the interaction between nutrients, gut microbes and host health is microbial-related inflammation process (for review, see de Lartigue et al [51]). The relationship between the gut microbiota, impact of a high fat diet and gut inflammation was investigated using germ-free mice fed a high-fat diet [52]. The latter exhibited neither an increase in TNFalpha mRNA nor activation of NF-κB in the ileum as compared to conventional mice, thereby suggesting that both the combination of high-fat diet and the presence of gut microbes are necessary to induce intestinal inflammation [52]. More importantly, the development of weight gain, adiposity, and plasma levels of insulin and glucose were positively associated with intestinal inflammation, further supporting the idea that intestinal inflammation may be an early event by which fat diets contribute or lead to the development of obesity and associated disorders [52]. In addition, specific components of bacterial membrane components have been implicated in the development of inflammation upon obesity and diabetes. This is the case of the lipopolysaccharides (LPS), the main component of the gram negative bacteria (for review, see [8]). The level of serum LPS is increased by about twice in obese, diabetic, or high fat diet fed individuals, by processes involving an increase in chylomicron (lipoproteins that transport triglycerides and cholesterol from the small intestine to other tissues) formation (upon high fat diet feeding), a decrease in gut barrier integrity, and a decrease in alkaline phosphatase activity, which is the enzyme responsible for the cleavage of the LPS in the intestine. Those metabolic alterations are illustrated in figure 2.

Other microbial derived elements play a role in the regulation of host homeostasis. Nutrients which escape the digestion to the upper part of the gut, are fermented by gut microbes, which release namely short chain fatty acids (SCFA) (acetate, propionate, butyrate). Those bacterial metabolites are absorbed by host intestinal tissue, and thereby could participate to “energy harvest”. In addition, SCFA are able to act as signalling molecules in host tissues, by linking selected G protein-coupled receptors, free fatty acid receptors FFAR2 and FFAR3 [53]. In fact, FFAR3^-/-^ mice, which do not express the receptor able to bind the SCFA, gain less weight when colonized with saccharolytic bacteria (*B. thetaiotaomicron* and *M. smithii*), as compared to mice expressing the receptor [54]. This suggests that the SCFA may promote weight gain by binding to this receptor. Other data have shown that SFCA (acetate, propionate) may stimulate adipogenesis in the white adipose tissue via FFAR2 activation [55].

These data suggest that the ingestion of fermentable carbohydrates de facto contribute to an increase in fat mass. Nevertheless, numerous other data obtained with non digestible/highly fermentable carbohydrates with prebiotic properties, counteract this hypothesis.

## Interaction of prebiotic carbohydrates with host energy metabolism

**Figure 3:**
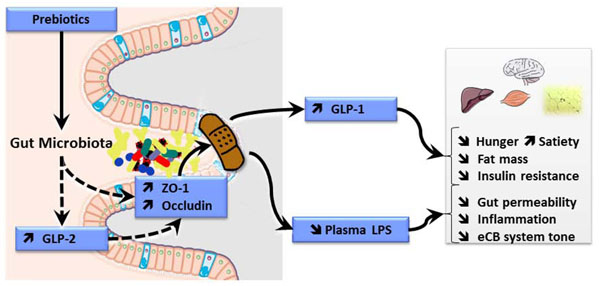
**Improvement of obesity and related metabolic disorders by the prebiotic approach**. Nutrients with prebiotic properties allows, by changing the gut microbiota, to promote the endocrine function of the gut (increase in GLP-1, and GLP-2 producing cells), and to modulate the activation of the endocannabinoid system in the intestine and in the adipose tissue. All those effects contribute to lessen gut permeability (improved distribution of the tight junction proteins ZO-1 and Occludin), thereby decreasing endotoxemia, and systemic inflammation. Changes in GLP-1 contribute to decrease food intake, fat mass, glycemia and insulin resistance. eCB, endocannabinoid; GLP-1, glucagon-like peptide 1; GLP-2, glucagon-like peptide 2; LPS, lipopolysaccharides; ZO-1, zonula occludens 1.

In different experimental models of obesity (*ob/ob* mice, diet-induced obesity, obese Zucker rats), dietary supplementation with non digestible/fermentable carbohydrates, such as inulin-type fructans or arabinoxylans, exert beneficial effects for the host and can reduce adiposity (see review, [9,56,57]). Dietary fructans have been more extensively studied as compared to other interesting compounds with potential prebiotic properties (galacto-oligosaccharides, xylooligosaccharides, lactulose) up to now, namely in the context of obesity [58]. Treating obese individuals with fructans-type prebiotics has been tried in a limited number of intervention studies. Ingestion of inulin-type fructans prebiotic (8g/d) for one year showed a significant benefit in the maintenance of BMI, and fat mass in non obese young adolescents [59]. The daily intake of yacon syrup, which delivered 0.14g fructans per kg per day over 120 days, increased satiety sensation and decreased body weight, waist circumference and body mass index in obese pre-menopausal women [60]. Finally, a recent clinical trial, whereby short chain inulin-type fructan were given as a supplement for 3 months (21g/d), decreased food intake, body weight gain and fat mass development in obese subjects, which supports the evidence that prebiotics promote weight maintenance [61]. The authors found higher plasma PYY levels following a meal, as well as a drop in ghrelin over a 6-hour meal tolerance test [61]. The modulation of gut peptides by fructans-type prebiotics (16g/d, two weeks) in healthy individuals also increased GLP-1, PYY and GIP, an effect which correlates with a decreased glycemic response, and a decrease in energy intake in healthy individuals supplemented with inulin type fructans for 2 weeks [62]. In obese animal fed inulin-type fructans (10% in the diet), a decrease in food intake through the modulation of the production of gastro-intestinal peptides also occurred, together with an increase in anorexigenic peptides (PYY and GLP-1 (7-36) amide) and a decrease in the orexigenic peptide ghrelin. This lead us to postulate that the improvement of obesity and related diseases by fermentable carbohydrates can be mediated through the modulation of the endocrine function of the gut. In fact, an increase in the number of L cells – linked to an increase in the differentiation of those cells in the jejunum and in the colon - is observed in rats and mice fed with inulin-type fructans prebiotics (for review, see [8]). By using models of mice lacking a functional GLP-1 receptor, it was demonstrated that the activation of the GLP-1 producing cells in the gut drives the improvement of glycemic and insulin response, lessens fat mass, and participates to the satietogenic effect of the prebiotics. On the other hand, the over-secretion of GLP-2 (co-secreted with GLP1 by L cells) is implicated in the lower systemic inflammation (decrease in circulating LPS and proinflammatory cytokines) occurring in obese mice (Figure 3). The decrease in LPS absorption through an improvement of the expression and activity of proteins involved in gut barrier function (Zonula-occludens 1 (ZO-1) and Occludin), occurs in prebiotics treated animals (Figure 3). Importantly, we found that changing the gut microbiota with prebiotics promotes the normalisation of the endocannabinoid system (eCB) responsiveness in the gut, thereby decreasing in gut permeability, metabolic endotoxemia and fat mass development (Figure 3). However, those effects are still unravelled in humans [49,56,62-68]. The “anti-inflammatory” effect of prebiotics seems particularly interesting, since as illustrated earlier, several studies suggest that the gut microbiota can be involved in the development of a low-grade inflammation classically associated with the metabolic disorders related to obesity [25][51,69].

The question of the relevance of specific gut microbial species/phyla in the improvement of metabolic syndrome by fructans-type prebiotics is a matter of debate. As previously described, several reports have shown that obesity induced following dietary manipulations (high fat feeding) or genetic deletion (leptin deficient models) is characterized by changes in gut microbiota towards a decreased number of bifidobacteria [70-75]. This group of bacteria has been shown to reduce intestinal LPS levels in mice and to improve the mucosal barrier function [25,40,49,76]. We can not preclude, in view of the complexity of the gut microbiota, that bifidobacteria are the sole actors in the improvement of metabolic disorders associated with obesity by prebiotics. Other non digestible/fermentable carbohydrates such as resistant starch (type 4), are able to increase Actinobacteria and specific types of bifidobacteria (*Bifidobacterium adolescentis*) in human individuals [77]. Those resistant starches have also been proposed as nutrients able to control glycemia and food intake by changing the profile of gut hormones, such as GLP-1. However, there is no proof until now that the increase in Bifidobacteria participates per se to the increase in the endocrine function of the gut upon prebiotics treatment.

In fact, we have recently studied the composition of the gut microbiota using the pyrosequencing of the 16S rRNA gene of the caecal content of ob/ob mice treated with prebiotics, and we discovered more than a hundred sequence which were different upon prebiotic treatment, some of them being modified by more than ten fold, some of them being correlated with the number of L cells in the colon and the intestine (PD. Cani, personal communication).

Altogether, those data imply that, if the gut microbial activity is devoted to ferment carbohydrates, the energy sparing due to the production of the short chain fatty acids may be supplanted by other biochemical events occurring in the gut upon the changes in the microbial composition and/or activity. In fact, we have recently shown upon fructans supplementation in high fat diet fed mice, that the increase in fermentation occurring in the caecum, leads to a decrease in the expression of the genes responding to the short chain fatty acids (FFAR2) in the adipose tissue , and thereby lessens the development of fat mass [78]. Adaptive processes thus take place far from the gut (for example in the liver and the adipose tissue) upon prebiotic approach, which could contribute to an improvement of metabolic syndrome. Those effects are illustrated in figure 3.

## Conclusion and perspectives

The elucidation of specific bacterial phyla/gender/species whose number correlates with fat mass development could help discovering a new type of “target” in the management of obesity and related disorders. Most of the data published until now in humans, have analyzed fecal samples in order to relate the changes in gut microbiota composition, obesity or food intake behaviour. In fact, the composition of the fecal microbiota does not fully represent the microbial composition in the colon. Moreover, recent data suggest that the mucosa of proximal small intestine is highly responsive to changes in the microbial composition driven by a probiotic approach [79]. In addition, we have shown that the modulation of the endocrine L cells number, induced in mice by the prebiotic approach, is occurring not only in the colon, but also in the jejunum. Finally, the events linking the host microbes to diabetes would also implicate the oral microbiota, which can be modified by prebiotics an probiotics [80,81]. All those data suggest that, even if the vast majority of microbes reside in the colon, one might also pay attention to microbial modulation occurring in other body compartments in order to understand how the interplay between nutrients and gut microbes influences host health, namely in the context of diabetes and obesity.

It is crucial to analyse the metabolic potential of the gut microbes in totally in order to understand the biochemical mechanism underlying the energy sparing or energy expenditure due to nutrients/microbial interaction. The saccharolytic activity of the gut microbiota, namely through the production of short chain fatty acids, has been proposed as a potential driver of adipogenesis. However, highly fermentable carbohydrates, such as prebiotics, are able to counteract most of the metabolic alterations linked to obesity, despite (or thanks to?) the fact that they are actively fermented into short chain fatty acids. It is likely that the targeted modification of the gut microbiota can also be obtained by the administration of bacteria themselves. For example, a targeted probiotic approach leading to the production of bioactive lipids prone to regulate host homeostasis (conjugated linoleic acids) are particularly interesting [82]. Finally, we largely ignore if and how the host itself may influence the composition of the gut microbiota, and thereby take advantage of its symbiotic partner. We have recently shown in mice deprived of dietary magnesium for 2 days that the magnesium deficiency promoted systemic and intestinal inflammation just within the two days [83]. However, those detrimental effects were abolished after three weeks of magnesium-deficiency, a phenomenon associated with specific changes in gut microbiota composition in favour of bacteria reinforcing the gut barrier function (increased bifidobacteria) [83]. Therefore, it’s possible that not only macronutrients, but also micronutrients and phytochemicals present in minor concentrations in the diet are interacting with the gut microbes to modulate host physiology. The relevance of this in the field of obesity and associated metabolic disorders remains to be unravelled.

The gut microbiota thus appears as an important target to consider in the management of obesity and related diseases. The advantage of this target is that both nutritional and pharmacological approaches will be developed upon the increasing knowledge of host-microbe interactions. The metabolomic approach will be without any doubt, a way to understand how microbial activities are related to host phenotype metabolism. The metabolomic NMR analysis of different tissues and fluids of conventional versus germ free mice allowed the presence of a specific family of bacteria (*Coriobacteriaceae* family) to be linked with the regulation of hepatic intermediary and xenobiotic metabolism [84]. Recently, it was shown by NMR analysis of biomarkers in the serum of genetically obese versus lean growing pigs that changes in gut microbiota-related metabolites occurred, including trimethylamine-N-oxide and choline, in addition to markers of lipogenesis, lipid oxidation, energy utilization and partition, protein and amino acid metabolism [85]. Future research needs to be performed to assess the relevance of those metabolites in host physiology homeostasis, a key question being: are there only markers or actors of the interactions between diet, host, and gut microbes?

Key words : gut microbiota, obesity, prebiotics, inflammation, gut peptides.

## Competing interests

The authors declare that they have no competing interests.
